# Bioaccumulation and potential sources of heavy metal contamination in fish species in River Ganga basin: Possible human health risks evaluation

**DOI:** 10.1016/j.toxrep.2019.05.012

**Published:** 2019-05-29

**Authors:** Pradip Kumar Maurya, D.S. Malik, Krishna Kumar Yadav, Amit Kumar, Sandeep Kumar, Hesam Kamyab

**Affiliations:** a Department of Zoology and Environmental Science Gurukula Kangari Vishwavidyalaya, Haridwar, 249404, India; b Institute of Environment and Development Studies, Bundelkhand University, Kanpur Road, Jhansi, 284128, India; c Department of Botany, Dayalbagh Educational Institute, Agra, 282005, India; d Centre for Environment Science and Climate Resilient Agriculture, Indian Agricultural Research Institute, New Delhi, 110012, India; e Engineering Department, Razak Faculty of Technology and Informatics, Universiti Teknologi Malaysia, Jalan sultan Yahya Petra, 54100, Kuala Lumpur, Malaysia

**Keywords:** Heavy metal, Fish, Bioaccumulation, Human health, Risk assessment, THQ

## Abstract

This paper assesses the potential human health risks posed by five heavy metals (Zn, Pb, Cu, Cd, and Cr) found in seven most consumable fish species (*Cirrhinus mrigala, Cirrhinus reba, Catla catla, Lebio rohita, Crossocheilus latius, Clupisoma garua, and Mystus tengara*) collected from local markets of Varanasi, Allahabad, Mirzapur, and Kanpur of Uttar Pradesh, India. The Cu concentration was found at Varanasi (4.58 mg/l), Allahabad (2.54 mg/l), and Mirzapur (2.54 mg/l). Pb was recorded 0.54, 0.62, 0.85, and 0.24 mg/l at Kanpur, Allahabad, Mirzapur, and Varanasi, respectively. The Cd concentration was recorded 0.54, 0.68, 0.78, and 0.85 mg/l at Kanpur, Allahabad, Mirzapur, and Varanasi, respectively. The Cr, Cd, and Pb concentrations in the river water were observed over the prescribed safe limits at all sampling sites, while Cu concentration was higher than the standards at all sites except Kanpur. However, Zn was observed under the permissible limits (15 mg/l) at all sampling sites. In case of fish tissues, WHO reported the concentration of Pb, Cd, and Cr higher than the prescribed safe limits. The results determined that the highest heavy metals accumulation was found settled in the liver of all selected fish species. Zn ranked the highest quantity, which was found in fish tissues with the concentration of 32.41 ± 2.55 μg/g in the gill of *C. catla* and 4.77 ± 0.34 μg/g in the gill *C. Reba*. The metals followed the magnitude order of Zn > Pb > Cu > Cd > Cr in selected fish tissues.

## Highlights

• Different pollution degrees have been evaluated to check the pollution level in the River.
• The concentration of all studied heavy metals in all fish samples were in the order of liver > gill > muscle.
• The liver and gills of *C. catla* and *C. mrigala* were found to have high concentration of Cu and Zn.
• There is no potential health risk due to the consumption of studied fish species as the THQ value is less than 1.
• Need special measures must be taken to decrease the health effects of human being near the catchment basin of river.

## Graphical abstract

**Figure:**
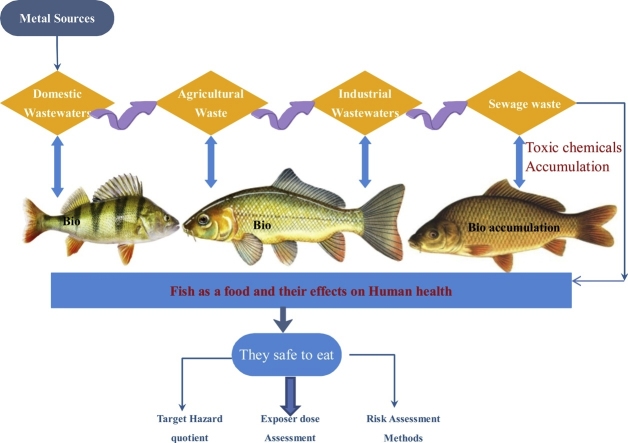

## 1 Introduction

The Ganga River (a perennial river originating from Gangotri glaciers), which is one of the major rivers of Ganga-Brahmaputra-Meghna system, contributes >43% (861,452 km^2^) of the cumulative catchment area. With an average annual running water potential of 525.02 Bm^3^ yr^−1^ (Billion cubic meters per year), which comes from all major Indian river basins [1], this river contributes substantially to Indian civilization and economy. The Ganga River biodiversity includes Phytoplankton and Periphyton (1099 taxa), Zooplanktons (299 taxa), zoobenthos (478 taxa), fishes (295 taxa), higher vertebrates (1595 taxa) [2]. Pollution, especially caused by partially treated and untreated waste, is the major threat to the river biodiversity. Partially treated and untreated waste is discharged into the river through about 36 Class-I towns and 14 Class-II towns. 2723.3 MLD (Millions of litter per day) wastewater is generated from these towns out of which 1208.8 MLD (40%) is mostly treated [3]. The maximum volume of wastewater is contributed by Uttar Pradesh (45 drains, 3289 MLD). The existence of heavy metals (Cd, Cr, Cu, Mn, Ni, Pb, and Zn) in the river water has been previously reported [[4], [5], [6], [7], [8]] together with sediments due to inputs of industrial wastes [9,10], sewage effluent [11], agricultural runoff, and domestic wastes [[12], [13], [14]]. However, the pedological processes also serve as the sources of pollutants, especially heavy metals that may appear due to the weathering of rocks through surface runoff water [[15], [16], [17]].

In addition, leachate from dumping sites can also cause surface water and groundwater pollution [[18], [19], [20]]. However, some of the pollutants are persistent due to their non-biodegradability and long biological half-life, e.g., heavy metals [[21], [22], [23]]. The distribution of heavy metals in water, sediments, and fish plays a key role in forming sources of heavy metal pollution in the aquatic ecosystem [24,25]. Pollution from domestic and industrial wastes is high at Kanpur and Allahabad, and Varanasi.

Fish is an important food of various inhabitant of the globe. Global per capita fish consumption has risen to above 20 kg year^−1^ [26]. Most of the people who live near banks of river are dependent on the fish as sources of protein. In India, annual per capita fish consumption is 5–6 kg for the general population and 8–9 kg for fish-eating population, which is about 50% of global consumption [27]. The present inland fish production contributes 6.57 million tonnes. The Ganga River contributes substantial fish production to its inhabitants. The *L. rohita, C. catla,* and *C. mrigala* fish species are abundantly found in this river. All the above-mentioned fish are the major sources of protein for human diet.

Health risks arising from the toxicity of metals mainly include kidney and skeletal damages, neurological disorders, endocrine disruption, cardiovascular dysfunction, and carcinogenic effects [28]. Dietary exposure to various heavy metals has been identified as a health risk to human through consumption of contaminated food. Many heavy metals bind with the sulfur present in enzymes, thereby disrupting their function [28,29]. Copper (Cu) may affect the gastrointestinal, cardiovascular, hematological, hepatic, renal, and CNS functioning. Zinc (Zn) can lead to vomiting, chest tightness, nausea, excitement, coldness, unconsciousness, and coma; even death may occur from pulmonary edema and liver damage. Higher Iron (Fe) intake can result in vomiting, diarrhea, gastrointestinal bleeding, metabolic acidosis, shock, hypotension, tachycardia, cardiovascular collapse, coagulation deficits, hepatic necrosis, and possibly death. Manganese (Mn) may cause dopaminergic dysfunction, neurochemical, neurobehavioral, neuroendocrine changes, and cardiovascular toxicity [30,31].

Some of these heavy metals are essential for the biological system and must be present within a particular concentration range. For example, iron (Fe), cobalt (Co), and manganese (Mn) are all needed by humans for various physiological and biochemical functions. Other heavy metals such as mercury (Hg), cadmium (Cd), lead (Pb), chromium (Cr), and Nickel (Ni) are toxic metals that can lead to contact dermatitis, lung fibrosis, cardiovascular and kidney diseases, as well as lung and nasal cancers [28,32,33].

For the purpose of this study, we collected both fish and water samples of upstream to downstream urban and city core of Varanasi, Allahabad, Mirzapur, and Kanpur. The specific objectives of the study were: 1) to assess the metal load of Cu, Zn, Fe, Mn, Ni, Pb, and Cd in muscles, gills, and liver tissue of the selected fish, 2) to estimate the potential health risk for consumers.

## 2 Materials and methods

### 2.1 Study area

**Fig. 1:**
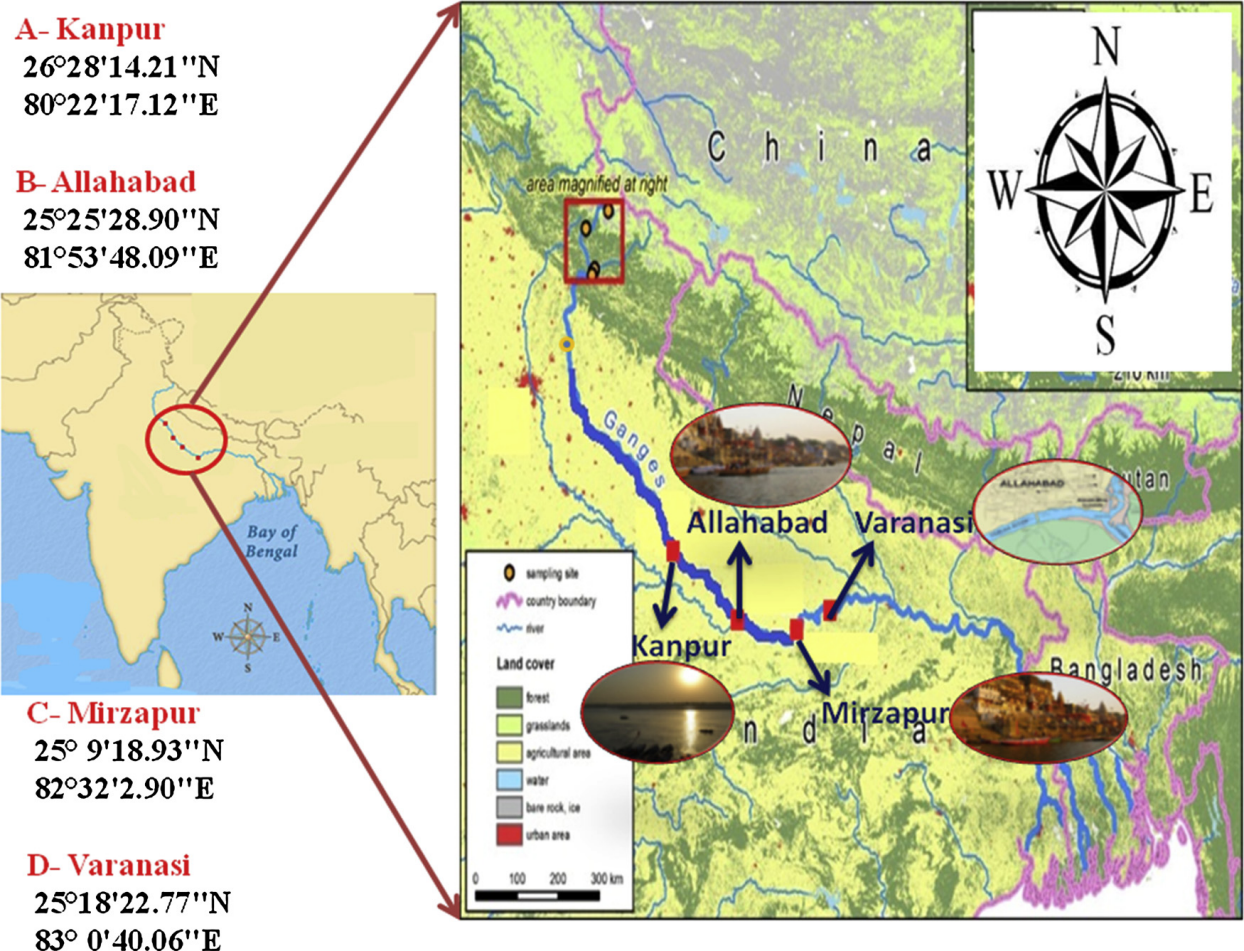
Sampling stations of the study sites.

The Ganga Plain is located in-between the Himalayan Mountain and Peninsular India. The plain occupies the central position of the Indo-Gangetic Plain and represents the world's largest, densely polluted alluvial plains. The Ganga River covers upstream to downstream urban and city core of Varanasi (site 1), Allahabad (site 2), Mirzapur (site 3), and Kanpur (site 4) (Fig. 1). In the sampling sites 1, 2, 3, and 4, numerous Indian industries and cities were found located on both sides of these rivers. The industrial effluents as well as domestic sewage/wastes are disposed of in these rivers either with partial or no pre-treatment, hence increasing concentration of different kinds of pollutants including heavy metals in the riverine water. The Ganga River region has long and hot summer (March-June), monsoon (June-September), and winter seasons (November-February). During the declining phase of the post-monsoon, these rivers deposit fine and very fine sand-dominated sediments in their active channel and flood plain areas, which frequently affect the agricultural practices and local population life.

### 2.2 Collection of samples

#### 2.2.1 Water samples

A total of 60 water samples were collected seasonally during the year 2016-17, including 20 samples from each season and 5 samples from each site. The total volume of 1 l water samples was collected in polyethylene bottles (twice rinsed with deionized water) and stored in an ice box and transported to the laboratory for further analysis.

#### 2.2.2 Fish samples

Seven sexually mature fish species on the basis of high consumption by the local population were collected from local fishermen and different dominant local markets of each study site, namely Varanasi, Allahabad, Mirzapur, and Kanpur during the year 2016-17 (Table 1). A total of 28 samples representing seven species from each individual study site were collected and wrapped in polyethylene bags, then an ice stored transportation was made to the laboratory for the biometrics, dissection, and collection of fish tissue for heavy metal analysis. In the laboratory, washing was performed with tap water for surface cleaning. After cleaning, tissue was isolated and chopped into small pieces using a stainless steel knife. Tissues were again cleaned with deionized water and air dried further to remove the extra water and debris; then they were homogenized in a food processor and 200 g of tissue were stored at −20 °C.

**Table 1:** The measurement of ecological characteristics and morphometric (biometrics) of selected fish species [34].

Scientific name	Common Name	Habitat	Feeding behaviour	Conservation status	No. of samples	Length (cm)	Weight (gm)
*C. mrigala*	Mrigal	Bottom feeder	Detritus feeder, vegetation, phytoplankton, and zooplankton	Least Concern	09-14	25-46	51.50-195.5
*C. reba*	Reba	Freshwater, benthopelagic, tropical	Plankton, detritus, vegetables, and insect larva feeder	Least Concern	11-17	23-30	45.08-58.5
*C. catla*	Catla	Surface and mid-water feeders	Mainly omnivorous	Least Concern	14-26	45-65	60.5-223.5
*L. rohita*	Rohu	Inhabits flowing and standing waters	Herbivorous, phytoplankton, and Zooplankton	Least concern	12-26	28-55	40.36-98.54
*C. latius*	Lurali	Preferably with the gravelly bottom in the benthopelagic environment.	Feeding on algae, diatoms and other phytoplankton	Least concern	10-25	09-15	32.06-50.55
*C. garua*	Guarchcha	Inhabit large freshwater and tidal rivers	Feed on insects, shrimps, other crustaceans and small fish	Least concern	17-25	36-52	52.47-124.8
*M.tengara*	Tengara	Middle and lower Ganga region (middle bottom feeder)	Carnivorous fish	Least concern	15-30	12-18	35.54-95.5

### 2.3 Instruments and reagents

A Varian AA240 atomic absorption spectrometer (AAS) with Zeeman background correction system equipped with a graphite furnace (GTA 120) was used to measure Cu, Zn, Fe, Mn, Ni, Pb, and Cd in the samples collected. The purity of standard and acetylene gases was 99.999% to 99.99%, respectively. Hollow cathode lamps were used for Zn (213.8 nm and slit 0.5), Pb (283.3 nm and slit 0.5 nm), Cu (324.75 nm and slit 0.5 nm), Cd (228.8 nm and slit 0.5 nm), and Cr (248.3 nm and slit 0.5 nm). The instrument was utilized according to the directions given by the manufacturer. Atomic signals for Zn, Pb, Cu, Cd, and Cr were measured in peak area mood. The digestions were performed using a hotplate (Model -Bio Technics BTI-22 9). All solutions were prepared in deionized water (18 MΩ/cm). All glassware and containers were cleaned by soaking into 20% nitric acid for 24 h and rinsed twice with deionized water prior to use.

### 2.4 Sample digestion

*Water:* 100 ml of filtered water samples were digested with concentrated HNO_3_ (20 ml) at 100 ^0^C. The digested water was cooled down to room temperature, diluted, and filtered through Whatmann-42 filter paper. The filtrate was made-up to 50 ml with 0.01 N nitric acid; then, the samples were ready for analysis.

*Fish:* 5 g identified tissue (dry) was digested in analytical grade HNO_3_:HClO_4_: HCl (3:2:9) for 4–6 hours on a hot plate. Next, the digested samples were cooled and filtered through the Whatman No. 42 filter paper. The samples were diluted up to 50 ml of distilled water for analysis.

### 2.5 Experimental analysis

Onsite measurement of the pH and temperature was performed using a portable meter. Dissolved oxygen (DO) and turbidity were observed using a DO data meter (Eutech CyberScan DO 3000) and multi-meter water checker (Horiba U-10), respectively, in Nephelometric units (NTUs). Total hardness (TH), total alkalinity, free CO_2_,and COD content were analyzed by the volumetric titration method [35].

The concentration of heavy metals in water sample was calculated using the following formula [36].

$$\text{H}\text{e}\text{a}\text{v}\text{y}\;\text{m}\text{e}\text{t}\text{a}\text{l}\;\text{c}\text{o}\text{n}\text{c}\text{e}\text{n}\text{t}\text{r}\text{a}\text{t}\text{i}\text{o}\text{n}\;\left(\text{μ}\frac{\text{g}}{\text{m}\text{l}}\right)=\frac{\text{A}\text{A}\text{S}\;\text{r}\text{e}\text{a}\text{d}\text{i}\text{n}\text{g}\;\times \text{V}}{\text{V}\text{o}\text{l}\text{u}\text{m}\text{e}\;\text{o}\text{f}\;\text{t}\text{h}\text{e}\;\text{s}\text{a}\text{m}\text{p}\text{l}\text{e}\;(\text{m}\text{l})\;}$$

where, V = volume of dilution solution

The concentration of heavy metals in fish tissue was calculated using the following formula:

$$\text{H}\text{e}\text{a}\text{v}\text{y}\;\text{m}\text{e}\text{t}\text{a}\text{l}\;\text{c}\text{o}\text{n}\text{c}\text{e}\text{n}\text{t}\text{r}\text{a}\text{t}\text{i}\text{o}\text{n}\;\left(\text{μ}\frac{\text{g}}{\text{m}\text{l}}\right)=\frac{\text{A}\text{A}\text{S}\;\text{r}\text{e}\text{a}\text{d}\text{i}\text{n}\text{g}\;\times \text{V}}{\text{W}\text{e}\text{i}\text{g}\text{h}\text{t}\;\text{o}\text{f}\;\text{t}\text{h}\text{e}\;\text{s}\text{a}\text{m}\text{p}\text{l}\text{e}\;(\text{g}\text{m})\;}$$

where, V = volume of diluted solution

### 2.6 Quality assurance and quality control

Calibration curve construction, quality assurance, and quality control were ensured considering different factors (blanks, calibration curve, spiked sample, and midpoint standard checks). Heavy metal analysis followed the Northern Ireland Environment Agency standards [37,38]. The calibration curve was guaranteed with the correlation coefficient (*R^2^*), where, Pb 0.9992, Cr-9999, Cu-9996, and Cd-0.9988. Mid-point checks for the metals lie in the range of 0.25 to 5.5%. Spike recoveries ranged from 96.54 to 98.85%.

### 2.7 Bioaccumulation factor

The bioaccumulation factors (BAF) are the ratio of heavy metals concentration in fish organ to that in water. BAF was determined using the formula suggested by Lau et al., (1998).

$$\text{B}\text{A}\text{F}=\frac{\text{C}\text{o}\text{n}\text{c}\text{e}\text{n}\text{t}\text{a}\text{r}\text{t}\text{i}\text{o}\text{n}\;\text{o}\text{f}\;\text{h}\text{e}\text{a}\text{v}\text{y}\;\text{m}\text{e}\text{t}\text{a}\text{l}\text{s}\;\text{i}\text{n}\;\text{f}\text{i}\text{s}\text{h}}{\;\text{c}\text{o}\text{n}\text{c}\text{e}\text{n}\text{t}\text{r}\text{a}\text{t}\text{i}\text{o}\text{n}\text{s}\;\text{o}\text{f}\;\text{h}\text{e}\text{a}\text{v}\text{y}\;\text{m}\text{e}\text{t}\text{a}\text{l}\text{s}\;\text{i}\text{n}\;\text{w}\text{a}\text{t}\text{e}\text{r}\;}$$

### 2.8 Quantitative health risk assessment

The fish muscles are mainly consumed by the human population as food. Therefore, we used fish muscles for evaluating the human health risk through an estimated daily intake (EDI) of metals and target hazard quotients (THQ).

#### 2.8.1 Estimated daily intake of metals

The estimated daily intake of heavy metals was calculated using the following equation.

$$\text{E}\text{D}\text{I}=\frac{\left(\text{C}\times \text{F}\text{I}\text{R}\right)}{\text{B}\text{W}}$$

where, C is the mean heavy metals concentration in fish muscle (μg/g) of dry weight basis. For conversion from dry weight to wet weight, 4.8 conversion factor is taken [39]. FIR (Food Ingestion Rate) is the daily consumption of freshwater fish (gram per day (g day^−1^) per capita. The average FIR was 0.019 g person^−1^ day^−1^ [26]. BW is the average body weight, 70 kg for adults [40].

#### 2.8.2 Target hazard quotient (THQ)

The THQ is the estimate of non-carcinogenic risk level due to heavy metals exposure [41]. It is calculated using the following equation [40].

$$\text{T}\text{H}\text{Q}\;=\frac{\text{E}\text{f}\text{r}\;\times \text{E}\text{D}\times \text{F}\text{I}\text{R}\times \text{C}\times 10¯3}{\text{R}\text{f}\text{D}\times \text{B}\text{W}\times \text{A}\text{T}\text{n}}$$

where Efr (Exposure frequency) is 365 d y^−1^, and ED (Exposure Duration) is 70 years (as set for this study). RfD (Reference Dose) assesses the health risk of consuming fish, and ATn is the time of average exposure for non-carcinogenic (365day × no. of exposure year) [40,42,43].

### 2.9 Statistical analysis

The data were statistically analyzed using the statistical package SPSS (version 16.0). The mean ± standard deviations of the metal concentration in fish species were calculated. Regarding the correlation coefficient level, if p < 0.05, it was evaluated as there was a statistically significant difference between the groups.

## 3 Results and discussion

### 3.1 Analysis of physicochemical parameters

The results of the physicochemical qualities of river water samples gathered from Kanpur, Allahabad, Mirzapur, and Varanasi sites are shown in Table 2. The temperature of the river water was observed in the range between 26.25–28.08 °C with an average temperature of 27.42 °C. Our observations are complying with approximately 50 year's previous results. This indicates that the temperature ranges are stable over time. The pH values of the samples ranged from 8.6 to 9.6 with a mean value of 8.96. In another study, the Ganga soil pH was observed ranging from 7.1 to 8.4 and Ganga water 7.0 to 9.2 with an average of 7.9 between the Kanpur and Patna. We found the lowest pH (8.6) at Kanpur and the highest pH (9.6) at Varanasi [44]. This might be due to the fact that more industrial effluent and sewerage water is drained at Kanpur region compared to the other sites. The increase in pH values of river water samples recorded from upstream to downstream indicated an increase in the pollution load from upstream to downstream. The pH values of water at sewage discharge points in the river were usually lower than that of the water taken from the other parts of the river.

**Table 2:** Physico-chemical parameters of the Ganga River water sample at different sites.

Parameters	Kanpur	Allahabad	Mirzapur	Varanasi	[48]
Tem (^o^C)	26.25 ± 0.21	27.35 ± 0.14	28.05 ± 0.41	28.08 ± 0.41	20-30
pH	8.6 ± 0.25	8.9 ± 0.18	9.3 ± 0.27	9.6 ± 0.21	6.5-8.5
Free CO2 (mg/l^−1^)	1.82 ± 0.08	3.23 ± 0.14	5.25 ± 0.12	5.85 ± 0.15	Nil
Total alkalinity (mg/l)	320 ± 5.14	370 ± 7.15	420 ± 10.25	470 ± 7.95	200
Total hardness (mg/l)	280 ± 3.14	298 ± 8.54	341 ± 10.50	391 ± 9.25	600
Turbidity	2.8 ± 0.05	3.15 ± 0.12	3.35 ± 0.32	3.56 ± 0.41	5
DO (mg/l)	8.54 ± 0.24	7.62 ± 0.29	7.36 ± 0.61	6.69 ± 0.38	–
BOD (mg/l)	18.64 ± 1.54	21.85 ± 2.45	22.18 ± 1.94	25.25 ± 2.24	–

The Ganga water has a strong buffering capacity but allies its water on the higher side of neutral pH as observed in the present study at four sampling location of the middle stretch. This indicates that water sample has an alkaline nature, which is not only slightly lethal to fish [45], but also imperfect for human health [31,46]. However, the European Union directed pH protection limits of 6.0 to 9.0 for fisheries and aquatic life [47]. If water turbidity is less than 5 NTU, according to Bureau of Indian Standards (BIS), the water is safe [48]. The total alkalinity was observed between 320–470 mg/l. The alkalinity of Ganga water is continuously increased due to the increase of the pollution load in the downstream from Kanpur to Varanasi. The high value of alkalinity indicates the presence of weak acid and strong base as carbonates, bicarbonates, and hydroxides in the water body [49].

The high volume of alkaline may also be due to the increase of free (CO_2_) in the Ganges River, which ultimately results in the rise of alkalinity at the Mirzapur site (Table 2). This condition may also occur because of the presence of strong bases such as carbonates, bicarbonates, and hydroxides in the water body [50]. The high values of alkalinity may also be due to a increase in free CO_2_ in the River Ganga by which bicarbonate ions are converted into carbonate, which ultimately results in an increase in alkalinity level at Mirzapur and Varanasi sites compared to Kanpur and Allahabad. Hard water refers to the water containing high levels of dissolved calcium, magnesium, and other mineral salts such as iron. The hardness levels varied from 280.5 to 391.2 mg/l with a mean value of 335.5 mg/l across the sampling location. The concentration of total hardness was very high in the selected site according to BIS (600 mg/l).

The DO measurement determines the purity of water. The amount of DO is a measure of the biological activity of the water masses and is widely used in water quality studies and routine operation of water reclamation facilities. In the present study, DO level of River Ganga of the selected site from January to December was fairly poor 6.69–8.54 mg/l with an average of DO 8.25 mg/l during the study. DO was found slightly decreased at Mirzapur and Varanasi sites due to different sewage additions of downstream. It was observed that DO concentration in Ganga River water is highly controlled by organic matter, depth, temperature, and turbulence. Since bacteria typically use DO in the process of decomposition, DO reaches the lowest level. A decrease in the DO volume from upstream to downstream was an indication of organic pollution load in the river; or it might be also due to increasing temperature.

The Biological oxygen demand (BOD) values varied from 18.64 to 25.25 mg/l during the study. During the present study, maximum BOD value 25.25 ± 2.24 mg/l was measured at Varanasi; the reason was that in this region, the sewerage line merged at the sampling location. The increased BOD in water may be due to the increase of organic pollution resulted from untreated domestic sewage, agriculture runoff, and residual fertilizers. CO_2_ plays a vital role in the life of plants and microorganisms. It is produced due to the respiration of aquatic organisms. The increased CO_2_ levels in the aquatic system is due to decay and decomposition of organic matter and addition of a large amount of sewage and respiration of aquatic plant, which is the main causal factor for an increase in CO_2_ in water bodies. The average free CO_2_ in the Ganga fluctuated between 1.82 and 5.85 mg/l^−1^ during the study. The free CO_2_ in the aquatic system is a balance of photosynthesis of autotrophs and respiration of autotrophs and heterotrophs. Generally, free CO_2_ is known as a dissolved gas. Surface waters normally contain < 10 ppm free CO_2_.

The heavy metals concentrations in the Ganga River water samples from four selected sites are presented in Table 3. The ranges of heavy metals concentration were recorded as follow: Cu: 1.35–4.58 mg/l; Zn: 4.74–8.44 mg/l; Pb: 0.24-0.85 mg/l; Cd: 0.54-0.85 mg/l, and Cr: 0.32-0.85 mg/l. The mean heavy metals loads in the Ganga River water of different sites were in the following order: Varanasi: Zn > Cu > Cd > Cr > Pb; Mirzapur: Zn > Cu > Pb > Cd > Cr; Allahabad: Zn > Cu > Cr > Cd > Pb; and Kanpur: Zn > Cu > Pb = Cd > Cr.

**Table 3:** Heavy metals concentration (mg/L) in River Ganga water at selected sites.

Heavy metals	Kanpur	Allahabad	Mirzapur	Varanasi	[56]
Cu	1.35 ± 0.25	2.54 ± 0.65	2.54 ± 0.68	4.58 ± 1.54	1.5
Zn	4.74 ± 0.14	5.25 ± 1.25	6.25 ± 3.54	8.44 ± 2.35	15
Pb	0.54 ± 0.05	0.62 ± 0.05	0.85 ± 0.08	0.24 ± 0.04	0.01
Cd	0.54 ± 0.07	0.68 ± 0.47	0.78 ± 0.12	0.85 ± 0.24	0.005
Cr	0.32 ± 0.06	0.85 ± 0.08	0.36 ± 0.07	0.45 ± 0.06	0.05

In this study, we found that all the selected heavy metals except the Zn were higher than the permissible limits stated by the World Health Organisation (WHO). The Ganga River water had the highest Cu (4.58 mg/l) at the Varanasi site followed by 2.54 mg/l at the Mirzapur and Allahabad sites. On the other hand, the lowest Cu concentration (1.35 mg/l) was observed at Kanpur (Table 3). The highest Pb concentration (0.85 mg/l) was found at the Mirzapur site followed by Allahabad (0.62 mg/l), while the lowest Pb concentration (0.24 mg/l) was observed at the Varanasi site. The highest (0.85 mg/l) and lowest (0.54 mg/l) levels of Cd concentrations were recorded at the Varanasi and Kanpur sampling sites, respectively. The highest and lowest Cr concentrations (0.85 & 0.32 mg/l) were observed at the Allahabad and Kanpur sites, respectively. The Cr concentrations at Varanasi and Mirzapur were found 0.45 mg/l and 0.36 mg/l, respectively. The Zn concentration was 8.44, 6.25, 5.25, and 4.75 mg/l at Varanasi, Mirzapur, Allahabad, and Kanpur, respectively. The Zn levels were recorded under the permissible limits at different sites.

The transport of heavy metals in the environment is highly controlled by the reactions of the metal with the water, sediments, and aquatic life forms and also their interaction with the other metals and environmental conditions [[51], [52], [53], [54]]. In [55], the author investigated the role of grain size distribution in the transport of lead, zinc, copper, and chromium; the other heavy metal loadings were found highly governed with the solid particles and their transportation through particulate matter in the aquatic ecosystem. The results of the present study indicated that anthropogenic waste, especially industrial effluent discharge and agricultural runoff, is released into the Ganga River, which cause water polluted seasonally with heavy metals; the accumulation of these persistent pollutants is a big risk for the fish.

### 3.2 Analysis of heavy metal concentrations in fish tissue

The concentration of heavy metals in the seven fish species was in the magnitude order of liver > gill > muscle. The fish muscles are majorly consumed as food across the globe. *C. mrigala and C. garua* fish species are major sources of protein and consumed throughout India. Thus, selected species were taken for in this study and also analyzed for different metals. The highest load of Zn was found in all the studied fish species followed by *L. rohita, M. tengara, C. garua, C. latius, C. mrigala, C. catla* and *C. reba*. The heavy metal concentration trend was Zn > Cu > Pb > Cd > Cr in almost all fish species. Findings of the present study also confirmed the results reported in [57,58]. However, the bioaccumulation magnitude is a species-specific function for trophic transfer [59].

In the present research, considerable variations were observed in the heavy metals concentrations among different species. Among the seven fish species, in cases of *L. rohita* and *C. catla*, the highest concentrations of almost all four metals were observed (Table 4). This was due to the larger size (higher biomass) of these species; larger fish tend to accumulate higher amount of heavy metals [60,61]. The lowest metals accumulation observed in *M. tengara* and *C. reba* might be due to their smaller body size, which reduces the accumulation of the metal through surface action [62]. In addition, this is probably due to the heavy metal concentration variation in the surrounding water medium along with the variation in the age of the selected fish species. In addition, metal speciation in the aquatic system, temperature, and pH need to be also considered importantly for metals accumulation [63,64].

**Table 4:** Concentrations of heavy metals (μg/g wet weight) in some organs of fish species collected from the Ganga River (Mean (±SD).

Fish species	Fish Tissues	Heavy metals				
		Cu	Zn	Pb	Cd	Cr
*C. mrigala*	Muscle	3.21 ± 0.54	11.25 ± 3.65	2.37 ± 0.21	1.32 ± 0.32	0.35 ± 0.11
	Gills	8.94 ± 2.62	17.54 ± 2.58	2.29 ± 0.35	1.85 ± 0.71	0.39 ± 0.05
	Liver	6.57 ± 0.54	25.08 ± 3.54	2.54 ± 0.05	2.64 ± 0.33	0.55 ± 0.22
*C. reba*	Muscle	0.58 ± 0.09	13.25 ± 1.22	3.89 ± 0.41	0.32 ± 0.07	0.28 ± 0.03
	Gills	0.85 ± 0.05	10.54 ± 2.60	4.77 ± 0.34	0.54 ± 0.03	0.83 ± 0.20
	Liver	2.55 ± 0.85	08.28 ± 1.22	1.54 ± 0.06	0.68 ± 0.11	0.33 ± 0.02
*C. catla*	Muscle	7.87 ± 2.58	15.24 ± 2.04	2.03 ± 0.11	0.65 ± 0.02	1.08 ± 0.06
	Gills	5.50 ± 0.55	11.25 ± 1.07	2.93 ± 0.51	1.25 ± 0.06	1.74 ± 0.31
	Liver	11.05 ± 2.65	18.25 ± 2.54	3.15 ± 1.22	1.32 ± 0.05	1.28 ± 0.42
*L. rohita*	Muscle	3.88 ± 0.15	25.36 ± 2.04	1.12 ± 0.03	0.65 ± 0.10	0.84 ± 0.05
	Gills	1.32 ± 0..4	32.41 ± 2.55	1.83 ± 0.06	0.82 ± 0.22	0.76 ± 0.12
	Liver	5.18 ± 1.99	28.97 ± 1.02	2.27 ± 0.22	0.74 ± 0.07	0.53 ± 0.06
*C. latius*	Muscle	1.27 ± 0.07	11.24 ± 0.91	1.27 ± 0.31	0.34 ± 0.6	1.20 ± 0.22
	Gills	2.59 ± 0.08	16.17 ± 1.55	1.54 ± 0.07	0.65 ± 0.24	1.02 ± 0.33
	Liver	3.54 ± 0.19	19.47 ± 2.91	2.85 ± 0.46	0.75 ± 0.07	1.54 ± 0.46
*C. garua*	Muscle	0.59 ± 0.04	18.34 ± 1.99	2.22 ± 0.22	0.52 ± 0.02	0.44 ± 0.03
	Gills	2.21 ± 0.62	25.22 ± 0.88	2.54 ± 0.06	0.68 ± 0.09	0.81 ± 0.08
	Liver	5.51 ± 1.09	29.98 ± 5.91	3.41 ± 1.02	0.98 ± 0.25	0.91 ± 0.12
*M. tengara*	Muscle	2.09 ± 0.14	21.45 ± 2.91	1.45 ± 0.06	0.39 ± 0.08	0.68 ± 0.07
	Gills	2.58 ± 0.33	28.63 ± 3.91	1.74 ± 0.09	0.45 ± 0.09	0.32 ± 0.03
	Liver	1.25 ± 0.02	40.29 ± 6.45	2.32 ± 0.74	0.85 ± 0.10	0.71 ± 0.04
[66]	Tissues	30	30	0.5	0.5	–
[89]	Tissues	30	40	0.5	–	0.15
[68]	Tissues	–	–	2.0	–	0.15
[69]	Tissues	–	–	0.2	0.5	–

#### 3.2.1 Copper

Copper (Cu) is an essential element for the formation of hemoglobin and some enzymes in human; however, high intakes can result in damage to liver and kidneys [65]. The highest Cu concentration was observed in the *C. catla* with 11.05 ± 2.65 μg/g in its liver, while the lowest concentration was found in *C. reba* with 0.58 ± 0.09 μg/g in its muscle. This indicates that Cu concentration had not exceeded the permissible limits suggested by international agencies such as Food and Agriculture Organization (FAO), World Health Organization (WHO) and Federal Environmental Protection Agency (FEPA) [[66], [67], [68]]. However, according to the Codex Committee on Food Additives and Contaminants (CCFAC), the continuous increase of Cu concentration in riverine ecosystem poses a seriously high health risk for human health through fish consumption [69]. In the Gangetic fish, Cu ranged between 0.02 ± 0.01 μg/g and 0.14 ± 0.05 μg/g in blood and 9.53 ± 0.31 μg/g and 31.62 ± 3.24 μg/g in muscles. Gills are directly exposed to water; thus, it is susceptible to the absorption of free divalent ions of heavy metals from acidic environment. High concentrations of Cu ions compete with other heavy metal ions for absorption through gills; thus, the bioavailability of Cu to fish increases [70,71].

#### 3.2.2 Zinc

Zinc (Zn) is an essential constituent of all living organisms for various enzymes such as carbonic anhydrase, transferrin, ferritin, and flavin iron enzymes. Zn was recorded as the highest concentration among the all heavy metals in all fish species in the four sites. The lowest (08.28 ± 1.22 μg/g) and highest (40.29 ± 6.45 μg/g) concentration of Zn was observed in the liver of *C. reba* and *M. tengara*, respectively. The highest Zn in the muscle (25.36 ± 2.04 μg/g) and gills (32.41 ± 2.55 μg/g) was observed in *L. rohita*; however, in the liver of *L. rohita*, Zn was recorded as high as 28.97 ± 1.02 μg/g. In another study, the Zn concentration in the muscles of *L*. *rohita* was recorded 32.24 ± 2.18 μg/g, 29.43 ± 0.74 μg/g, and 29.47 ± 2.47 μg/g in Rampur, Shivpuri, and Khajoorgaon, respectively. Zn concentration according to FAO, 30 μg/g recommended for the effluent of a dominated rivulet in India [66,69]. In the Gangetic fish (*C*. *Striatus; L*. *rohita and* C. *batrachus*), Zn ranged from 19.42 ± 1.49 μg/g to 41.06 ± 4.26 μg/g in muscles. The highest Zn (41.06 ± 4.26 μg/g) concentration was observed in muscles of carnivorous fish *C. striatus* [72]. In [73], the Zn concentration was recorded 08–40.29 μg/g in case of the Gangetic fish. The authors in [74] observed relatively high content of Zn (135.6 μg/g) in *Penaeus indicus*. The current results of a heterogeneous pattern of heavy metal accumulation in fish tissues might be due to the feeding behaviour of fish species [75].

#### 3.2.3 Lead

The lead (Pb) concentration ranged from 1.12 ± 0.03 to 4.77 ± 0.34 μg/g among the fish selected from the study area. The highest Pb concentration was detected 4.77 ± 0.34 μg/g in gill, for *C. reba* and 3.15 ± 1.22 μg/g in liver for *C. catla,* while the length and weight of both fish species were higher than the other selected fish species. The FAO and WHO proposed a limit of 0.5 μg/g for Pb in food, while FEPA set this value to 2.0 μg/g. The larger fish (*C. catla*, *L. rohita*, and *C. mrigala*) tend to accumulate more heavy metals due to extensive column feeding nature [17,66,76]. They have an increase in the metal accumulation through feeding quantity and surface action. On the other hand, the lowest accumulations were recorded for *C. reba*, *C. garua*, and *M. tengara*, which was due to their smaller body size [77]. Metal accumulation in *L. rohita* was investigated similar to *P. sophore* studied in by other researchers in other rivers [77].

#### 3.2.4 Cadmium

Cadmium (Cd) is a serious contaminant and a highly toxic element, which is transported in the water and air and found in different sources. The Cd concentration ranged from 0.32 ± 0.07 to 2.54 ± 0.33 in the selected fish tissues. The high load of Cd in the Ganga River is due to different industrial and domestic channels induced in the Ganga River. The maximum concentration of Cd (2.54 ± 0.05) was detected in the liver of *C. mrigala* and also 0.53 ± 0.13 μg/g to 1.42 ± 0.23 μg/g in muscles.

The highest volume of Cd was recorded by the authors in [78] as 1.42 ± 0.23 μg/g in muscles of carnivorous fish *C. striatus*. Vannoort and Thomson observed a lower Cd concentration (compared to the present study) varied from 0.003-0.036 mg/kg with a mean of 0.01367 mg/kg in vacuum packaged smoked fish species (*Mackerel*, *S. salar*, *and O. mykiss*) [79]. For instance, a study in canned tuna fish observed Cd concentration between 0.08-0.66 mg/kg, which is also higher than findings of this study [80]. In another study conducted on seasonal Cd concentration in the fish and oysters of the Shitalakhya River, Bangladesh, the amount was reported ranging between 1.09 and 1.21 mg/kg [77].

#### 3.2.5 Chromium

The chromium (Cr) concentration among the selected fish tissue ranged from 0.28 ± 0.03 to 1.74 ± 0.31. The lowest levels of the chromium concentration in muscle were recorded as 0.28 ± 0.03 μg/g in *C. reba*, 0.35 ± 0.11 μg/g in *C. mrigala*, and 0.44 ± 0.03 μg/g in *C. garua*, respectively. European Union Commission, suggested the daily tolerable chromium concentration to be 1 μg/g, while the FEPA suggested 0.15 μg/g and WHO suggested 0.15 μg/g. Earlier reports in regard to the Cr concentrations from the southeast coast of India indicated the range of 0.41–1.56 μg/g and 0.65–0.92 μg/g [81,82].

The Cr concentration in the present study was almost similar to *E. suratensis in muscle* [83]. The source of Cr could be attributed to agricultural runoff, paints used in boats, and leaching from rocks in the study area [84,85]. In a study into metal content in the fish in the Rishikesh to Kolkata stretch of river Ganga [85], the contents of Cr, Cu, Pb, and Zn were found high in the fish samples collected from the middle stretch of the river. The high levels of Cr and Pb have been previously found in river water and fish tissues of the Ganga River, which poses a great risk to the fish [86,87]. In case of the Gangetic fishes, Mn, Pb, and Zn concentrations in muscles are higher than the concentrations of Cd, Cu, Cr, and Ni [88].

### 3.3 Correlation analysis of heavy metal in fish tissue

Table 5 shows the relation of the elements through Pearson's correlation matrix. There is only one remarkable correlation between Cd and Cu (r = 0.75, p < 0.05). This is probably due to the high concentration of these two elements in *C. catla* and *C. mrigala* at all selected fish organs including other heavy metals. The accumulation of Cd and Cu was reported to occur due to the waste of electroplating, petrochemical, production, and chemical-intensive industries [90,91]. Accumulation of Cd and Cu by *C. catla* and *C. mrigala* had already been observed in other studies [14]. The negative correlation was calculated in case of Zn to Cu, Pb, Cd and Cr; and Cr to Pb and Cd. Moreover, in case of Pb to Cu, Cd to Cu, Cr to Cu, and Cd to Pb, there were significant positive correlations (p < 0.05) in between in the polluted water, which showed significant negative relationships with the gill and muscle inversely.

**Table 5:** Inter-elemental correlation matrix of heavy metals in the fish of the river Ganga.

Heavy metals	Cu	Zn	Pb	Cd	Cr
Cu	1				
Zn	−0.098	1			
Pb	0.654	−0.509	1		
Cd	0.757	−0.223	0.407	1	
Cr	0.271	−0.244	−0.008	0.090	1

### 3.4 Determination of bio-concentration factor

**Fig. 2:**
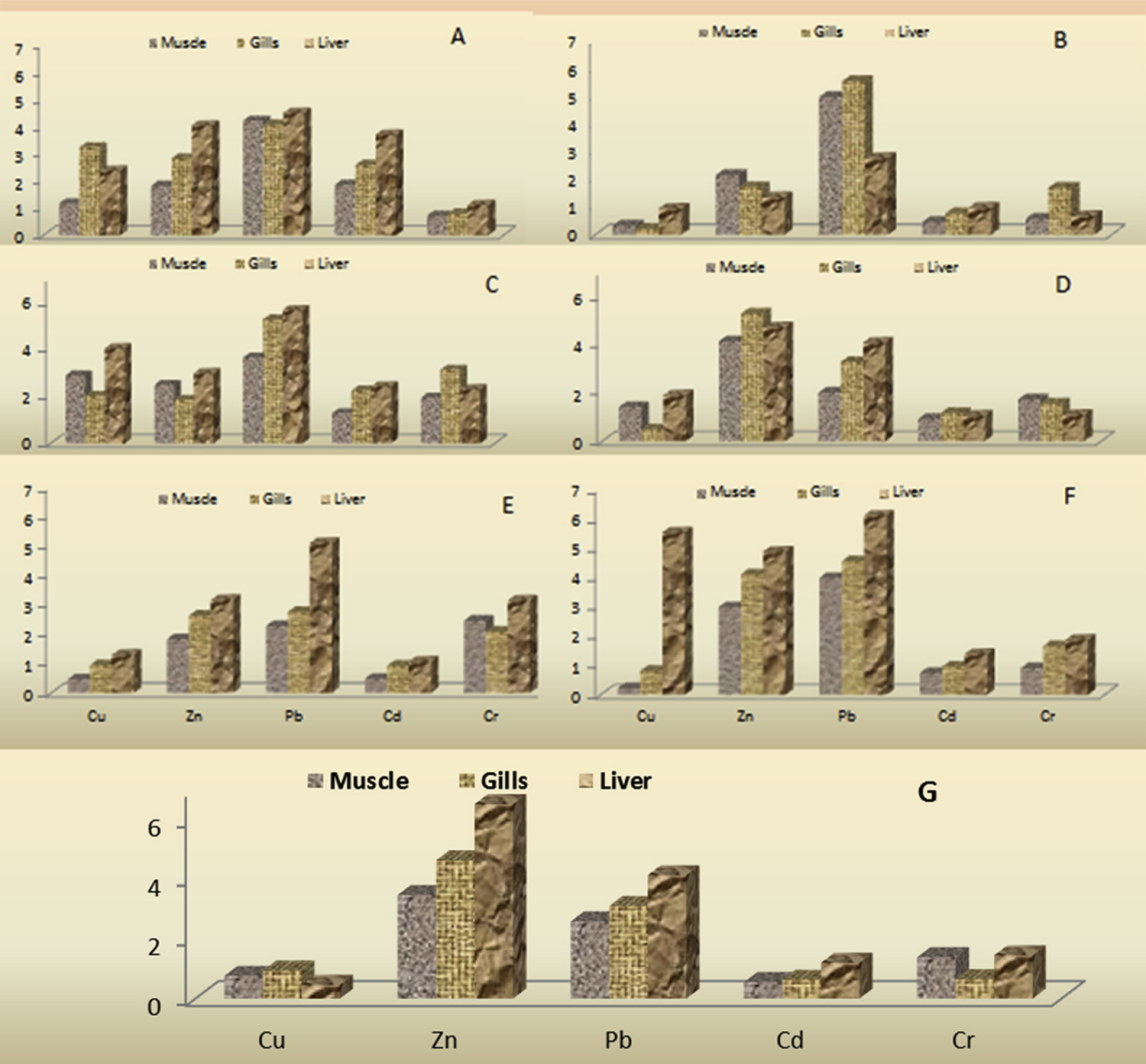
BCF for different metals in tissues of fish collected from the Ganga River (A) *C. mrigala*, (B) *C. reba*, (C) *C. catla*, (D) *L. rohita*, (E) *C. latius*, (F) *C. garua*, and (G) *M. tengara.*

Bio-concentration factors (BCFs) of heavy metals in fish tissues are the ratio of the heavy metals in tissue to surrounded water [92]. In the present study, the BCF of the heavy metals in the species-specific different fish tissues, i.e., gill, liver, and muscle showed that there was an appreciable chance of bioaccumulation of the different heavy metals in the fish body organ tissues. The liver of each fish species showed a higher BCF, while gill and muscle showed a lower BCF value. It was indicated that the concentration of heavy metals was transferred through the water to tissues of all the selected fish. The BCF in the present study showed that the concentration of the metals in the tissues followed the order of liver > gill > muscle. BCFs magnitude ranking was as follows: Cr, Cu, Cd, Zn, and Pb (see Table 6 and Fig. 2(A–G). Metabolically active tissues, i.e., gills, liver, kidneys, and showed higher accumulations of heavy metals than other tissues such as skin and muscles [93].

**Table 6:** Bio-concentration factor (BCF) index of the selected fish in different heavy metals.

Fish species	Fish Tissues	Cu	Zn	Pb	Cd	Cr
*C. mrigala*	Muscle	1.167	1.823	4.232	1.859	0.714
	Gills	3.250	2.842	4.089	2.598	0.795
	Liver	2.389	4.064	4.535	3.718	1.122
*C. reba*	Muscle	0.321	2.147	4.946	0.450	0.571
	Gills	0.210	1.708	5.517	0.760	1.693
	Liver	0.927	1.341	2.750	0.957	0.673
*C. catla*	Muscle	2.861	2.470	3.625	1.267	1.928
	Gills	2.00	1.823	5.232	2.232	3.107
	Liver	4.018	2.957	5.625	2.410	2.285
*L. rohita*	Muscle	1.410	4.110	2.000	0.915	1.714
	Gills	0.480	5.252	3.267	1.154	1.551
	Liver	1.883	4.695	4.053	1.042	1.081
*C. latius*	Muscle	0.461	1.821	2.267	0.478	2.448
	Gills	0.941	2.620	2.750	0.915	2.081
	Liver	1.287	3.155	5.089	1.056	3.142
*C. garua*	Muscle	0.214	2.972	3.964	0.732	0.897
	Gills	0.803	4.087	4.535	0.957	1.653
	Liver	5.51	4.858	6.089	1.380	1.857
*M. tengara*	Muscle	0.760	3.476	2.589	0.549	1.387
	Gills	0.938	4.640	3.107	0.633	0.653
	Liver	0.454	6.529	4.142	1.197	1.448

### 3.5 Health risk assessment

**Fig. 3:**
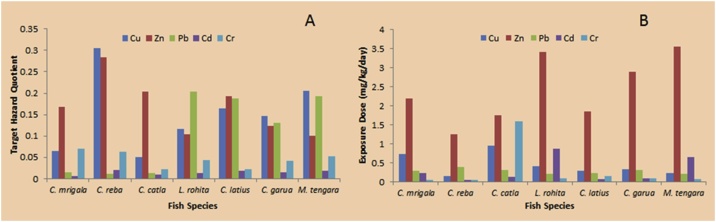
A: Target hazard quotient (THQ); 3B) Exposure Dose of heavy metals through consumption of the fish of the Ganga River basin.

The accumulation of heavy metals in the fish could affect directly the health conditions of the consumers living both in and outside the fishing site and consuming the fish on a daily basis. Therefore, the health risk assessment is essentially needed for fishes coming from contaminated resources. The health risk assessments, which are conducted based on the assumption of the most chemicals with noncancerous effects, exhibit a threshold response [94]. The Target Hazard quotient (THQ) estimated for individual heavy metals through consumption of different fish species are presented in Table 7 and Fig. 3A. The exposure dose of heavy metals through the consumption of fish from the Ganga River basin is given in Fig. 3B. The acceptable guideline value for THQ is 1 [40].

**Table 7:** The consumption of contaminated fish by human beings from the Ganga River and its effects calculated for different statistical analyses through estimation of daily intake (EDI) and target quotient (THQ), RfD = recommended doses of heavy metals as established by the United States Environmental Protection Agency [40,43].

Fish species	Heavy metals	Average concentration	Recommended daily allowance mg day^−1^ 70 kg^−1^ body weight	EDI 70 kg^−1^ body weight	RfD μg/kg^−1^ day^−1^	Target hazard quotient (THQ)
*C. mrigala*	Cu	6.24	35	0.736	0.040	0.0647
	Zn	17.95	70	2.198	0.3	0.1686
	Pb	2.40	0.25	0.283	0.0035	0.0147
	Cd	1.93	0.07	0.227	0.001	0.0053
	Cr	0.43	0.23	0.050	0.003	0.0704
*C. reba*	Cu	1.326	35	0.156	0.040	0.3044
	Zn	10.69	70	1.261	0.3	0.2832
	Pb	3.40	0.25	0.401	0.0035	0.0103
	Cd	0.513	0.07	0.060	0.001	0.0196
	Cr	0.48	0.23	0.056	0.003	0.0630
*C. catla*	Cu	8.14	35	0.960	0.040	0.0496
	Zn	14.90	70	1.759	0.3	0.2032
	Pb	2.70	0.25	0.318	0.0035	0.0130
	Cd	1.07	0.07	0.126	0.001	0.0094
	Cr	1.36	0.23	1.600	0.003	0.0222
*L. rohita*	Cu	3.46	35	0.408	0.040	0.1167
	Zn	28.91	70	3.411	0.3	0.1047
	Pb	1.74	0.25	0.205	0.0035	0.2030
	Cd	0.73	0.07	0.868	0.001	0.0137
	Cr	0.71	0.23	0.083	0.003	0.0427
*C. latius*	Cu	2.466	35	0.291	0.040	0.1637
	Zn	15.62	70	1.843	0.3	0.1938
	Pb	1.886	0.25	0.222	0.0035	0.1873
	Cd	0.58	0.07	0.068	0.001	0.0174
	Cr	0.252	0.23	0.147	0.003	0.0222
*C. garua*	Cu	2.77	35	0.326	0.040	0.1457
	Zn	24.51	70	2.890	0.3	0.1235
	Pb	2.72	0.25	0.320	0.0035	0.1298
	Cd	0.726	0.07	0.085	0.001	0.0139
	Cr	0.72	0.23	0.084	0.003	0.0420
*M. tengara*	Cu	1.973	35	0.232	0.040	0.2048
	Zn	30.123	70	3.554	0.3	0.1005
	Pb	1.836	0.25	0.216	0.0035	0.1924
	Cd	0.563	0.07	0.660	0.001	0.0179
	Cr	0.570	0.23	0.067	0.003	0.0530

The intake of heavy metals-contaminated freshwater fish has a high concern for human health [95,96]. The estimated daily intakes of Cu, Zn, Pb, Cd, and Cr were below the guideline reference doses of 0.040, 0.3, 0.0035, 0.001, and 0.003, respectively [40,43]. Consequently, the presence of Cd, Cr, Pb, Cu, and Zn in the edible tissues of the different fish species of Ganga River may not pose any serious human health risk after consumption.

## 4 Conclusion

The finding of the present study was compared with national and international standards (BIS and WHO) for drinking water, and it was found that the Ganga River water is not suitable for consumption without proper treatment at the selected sites. The Cu concentration was 4.58 mg/l at Varanasi, while it was 2.54 mg/l at the Allahabad and Mirzapur sites. Pb was 0.54 mg/l at the Kanpur site, 0.62 mg/l at Allahabad, 0.85 mg/l at Mirzapur, and 0.24 mg/l at Varanasi. The Cd concentration was observed 0.54, 0.68, 0.78, and 0.85 mg/l at Kanpur, Allahabad, Mirzapur, and Varanasi, respectively. The Cr, Cd, and Pb were observed over the prescribed safe limits at all sampling sites. Cu was higher at all sites except Kanpur. Zn was observed under the permissible limits (15 mg/l) at all sampling sites. The toxic metals were found accumulated in muscle, gill, and liver, where the highest concentration found in the liver. The high carcinogenic risk for consumers related to Cd, Cr and Pb were found out of permissible limits. Although Estimation of daily intake (EDI) calculation of heavy metals concentration is less than the recommended daily allowance. The heavy metals concentrations in the fish living in the Ganga River were considerably higher than the safe limits suggested by WHO and FAO.

According to BAFs of Pb, Cd and Zn are most readily absorbed and bioaccumulation heavy metals in the River Ganga fishes. The THQ was not more than 1 for in all fish species. The bioaccumulation of heavy metals in edible fish species may be considered as a warning for the negative impacts of fish consumption on human health. The present study shows that effective precautionary measures need to be taken in order to prevent future metal contaminants in the Ganga River water. Heavy metals contamination in the fish stock of the Ganga River must motivate imperative, urgent, and corrective actions from all the responsible parties to not only prevent and mitigate the situation, but also protect the well-being of local inhabitants significantly.

## Conflict of interest

The authors declare that they have no conflict of interest.
